# STIM1, ORAI1, and KDM2B in circulating tumor cells (CTCs) isolated from prostate cancer patients

**DOI:** 10.3389/fcell.2024.1399092

**Published:** 2024-06-05

**Authors:** Argyro Roumeliotou, Saad Alkahtani, Saud Alarifi, Abdullah A. Alkahtane, Christos Stournaras, Galatea Kallergi

**Affiliations:** ^1^ Laboratory of Biochemistry/Metastatic Signaling, Section of Genetics, Cell Biology and Development, Department of Biology, University of Patras, Patras, Greece; ^2^ Department of Zoology, College of Science, King Saud University, Riyadh, Saudi Arabia; ^3^ Department of Biochemistry, Medical School, University of Crete, Heraklion, Greece

**Keywords:** KDM2B, calcium signaling, ORAI1, STIM1, SOCE, prostate cancer, circulating tumor cells

## Abstract

**Introduction:** Previous publications have shown that STIM1, ORAI1, and KDM2B, are implicated in Ca^2+^ signaling and are highly expressed in various cancer subtypes including prostate cancer. They play multiple roles in cancer cell migration, invasion, and metastasis. In the current study we investigated the expression of the above biomarkers in circulating tumor cells from patients with metastatic prostate cancer.

**Methods:** Thirty-two patients were enrolled in this study and CTCs’ isolation was performed with Ficoll density gradient. Two different triple immunofluorescence stainings were conducted with the following combination of antibodies: CK/KDM2B/CD45 and CK/STIM1/ORAI1. Slides were analyzed using VyCAP microscopy technology.

**Results:** CTC-positive patients were detected in 41% for (CK/KDM2B/CD45) staining and in 56% for (CK/STIM1/ORAI1) staining. The (CK+/KDM2B+/CD45–) and the (CK+/STIM1+/ORAI1+) were the most frequent phenotypes as they were detected in 85% and 94% of the CTC-positive patients, respectively. Furthermore, the expression of ORAI1 and STIM1 in patients’ PBMCs was very low exhibiting them as interesting specific biomarkers for CTC detection. The (CK+/STIM1+/ORAI1+) phenotype was correlated to bone metastasis (*p = 0.034)*, while the (CK+/STIM1+/ORAI1–) to disease relapse (*p = 0.049)*.

**Discussion:** STIM1, ORAI1, and KDM2B were overexpressed in CTCs from patients with metastatic prostate cancer. STIM1 and ORAI1 expression was related to disease recurrence and bone metastasis. Further investigation of these biomarkers in a larger cohort of patients will clarify their clinical significance for prostate cancer patients.

## 1 Introduction

The second most frequent cancer for men, on a worldwide scale, is prostate cancer (Sung et al., 2021). When diagnosed at early stages the disease is relatively manageable however, at tumor progression, patients may gain resistance to treatment and create metastatic lesions (Sharma et al., 2018). Prostate cancer patients with distant metastasis, have aggressive disease with poor survival (Sharma et al., 2018; Zhang et al., 2022), justifying prostate cancer as the second leading cause of cancer mortality for men in the western world (Zhou et al., 2017). Thus, it is crucial to identify new targets for treatment to overcome acquired resistance to therapy and metastasis.

CTCs are the major contributors to the disease progression and the initiation of metastatic dissemination, as they can intravasate blood vessels, travel through the bloodstream, and extravasate from the vessels to colonize other tissues and organs (van Zijl et al., 2011). Most CTCs entering the bloodstream, are apoptotic, however, a subset of them can create metastasis (Kallergi et al., 2013). The detection of CTCs is a rather difficult process (Lianidou et al., 2014; Heidrich et al., 2021; Pantazaka et al., 2021), because tumor cells to detach from the primary tumor and migrate into the bloodstream must lose their epithelial characteristics and acquire mesenchymal features, through a process called epithelial-mesenchymal transition (EMT) (Kallergi et al., 2011; Nieto et al., 2016; Satelli et al., 2017). Therefore, there is an urgent need to identify these aggressive subclones of CTCs, that are responsible for generating metastases, through their expression of distinct biomarkers (Kallergi et al., 2013).

Specific intracellular signal transduction pathways promote Ca^2+^ to be released from the endoplasmic reticulum (ER) stores. For normal cells to retain their homeostasis, store-operated calcium entry (SOCE) is used for Ca^2+^ intracellular control (Xu et al., 2015; Zhang et al., 2017). SOCE is an ion channel consisting of two important components, stromal-interacting molecule 1 (STIM1) and calcium release-activated calcium channel protein 1 (ORAI1). When Ca^2+^ levels decrease, STIM1 as a calcium sensor, aggregates and moves to membrane areas interacting with pores formed from ORAI1. STIM1 mediates ORAI1 channel activation to promote Ca^2+^ influx through SOCE (Zhang et al., 2017). SOCE is the major influx of Ca^2+^ in non-excitable cells (Zhou et al., 2017), however, high expression of STIM1, ORAI1, and activated SOCE were found to be involved in cancer progression, migration, and metastasis for cervical cancer (Chen et al., 2011; Chen et al., 2013), breast cancer (Yang et al., 2009) and hepatocarcinoma (Yang et al., 2013).

According to previous publications from our group and other research teams, STIM1 and ORAI1 are implicated in metastasis of prostate cancer, as it has been shown to promote invasion, migration, and EMT in prostate cancer cells (Xu et al., 2015; Stagno et al., 2017; Zhou et al., 2017). We have recently shown that ORAI1 and STIM1 expression was controlled by KDM2B which is a histone demethylase belonging to the F-box protein family, regulating phosphorylation-dependent ubiquitination. KDM2B is a novel oncogene, and it is upregulated in a wide range of cancers. It is implicated in SOCE calcium signaling, actin cytoskeleton rearrangement, and cell migration (Zacharopoulou et al., 2018a; Zacharopoulou et al., 2018b).

Since CTCs are the major players in metastasis in the current study, we investigated for the first time the expression of STIM1, ORAI1, and KDM2B simultaneously in Circulating Tumor Cells from the same blood draw of metastatic prostate cancer patients. We also correlated our results with clinical data to identify potential aggressive CTCs’ subclones and evaluate the clinical relevance of the aforementioned biomarkers.

## 2 Materials and methods

### 2.1 Patients’ characteristics, patients’

A total of 32 metastatic prostate cancer patients were enrolled in the study. The patients’ median age was 72 years old (range: 42–92) (Table 1).

**TABLE 1:** Patients’ characteristics.

Variable	Sub-categories n (%)
Age	
≥75	13 (41%)
<75	9 (28%)
Unknown	10 (31%)
Gleason score, n (%)	
≥8	7 (22%)
<8	3 (9%)
Unknown	22 (69%)
Mean PSA (Range), (ng/mL)	72 (2–314)
Smoking Consumption	
Yes	16 (50%)
No	11 (34%)
Unknown	5 (16%)
Resistance to androgen deprivation treatment	
Hormone sensitive	14 (44%)
Castration resistant	8 (25%)
Unknown	10 (32%)
Status	
Metastatic	32 (100%)
Metastasis locations	
Bone metastasis	12 (38%)
Lymph node metastasis	5 (16%)
Lung metastasis	3 (9%)
Bone marrow metastasis	1 (3%)
Unknown	16 (50%)
Chemotherapy	
Baseline	30 (94%)
After 1st line (Docetaxel)	2 (6%)
Progression Disease	
Relapse	5 (16%)
No Relapse	19 (59%)
Unknown	8 (25%)

Peripheral blood from all patients and 10 healthy donors, was collected in EDTA K2 tubes. All blood samples were collected in the middle of the vein puncture. The first 5 mL were discarded to avoid contamination with epithelial cells from the skin. The protocol was approved by the Ethics and Scientific Committee of the involved institution Larissa General University Hospital, (32710/3-8-20). Patients and healthy donors gave their written informed consent for their participation in the study. Additionally, patients gave consent for their clinical follow-up data to be used for research purposes.

### 2.2 Blood collection and cytospin preparation

Peripheral blood mononuclear cells (PBMCs) from prostate cancer patients and healthy donors were isolated using Ficoll-Hypaque (d = 1.077 g/mol) density centrifugation at 1800 rpm for 30 min without brakes. PBMCs were washed twice with PBS and centrifuged at 1,500 rpm for 10 min. Aliquots of 500,000 cells/500 μL were centrifuged at 2000 rpm for 2 min on Superfrost glass slides (Thermo Fisher Scientific, Waltham, MA, United States). Cytospins were dried up and stored at −80°C. CTCs precluded in the fraction of mononuclear cells after Ficoll-Hypaque density centrifugation. Ficoll-Hypaque density gradient is an EpCAM-independent and commonly used method to isolate CTCs. This method enables the detection of both CTCs with epithelial and EMT features such as EpCAM-negative tumor cells (Nieto et al., 2016). Ficoll density gradients was used in many previous publications of our group (Kallergi et al., 2011; Kallergi et al., 2019; Roumeliotou et al., 2022; Vardas et al., 2023).

### 2.3 Cancer cell culture and spiking experiments

PC-3 and DU-145 cell lines obtained from the American Type Culture Collection (Manassas, VA, United States) were used for the development of the staining protocol regarding STIM1, ORAI1, and KDM2B expression. PC-3 and DU-145 cells were cultured in Dulbecco’s Modified Eagle Medium with Glutamax (Thermo Fisher Scientific, Waltham, MA, United States) supplemented with 10% fetal bovine serum (FBS; PANBiotech, Germany), and 50 U/mL penicillin/50 g/mL streptomycin (Thermo Fisher Scientific, Waltham, MA, United States). Cells were maintained at 37°C in a humidified atmosphere of 5% CO_2_ in the air and sub-cultivation was performed with 0.25% trypsin-EDTA (Thermo Fisher Scientific, Waltham, MA, United States).

For spiking experiments, PC-3 and DU-145 cells were spiked into healthy volunteers’ PBMCs (1000 H1299 cells/100,000 PBMCs). Spiked cancer cells and PBMCs were suspended in a solution of PBS with 2% FBS and centrifuged at 2,000 rpm for 2 min on Superfrost glass slides (Thermo Fisher Scientific, Waltham, MA, United States). Slides from spiking experiments were used as positive and negative controls in triple immunofluorescence stainings, by omitting one primary antibody in each negative control to evaluate the sensitivity and specificity of the method and the crosstalk between the corresponding antibodies.

### 2.4 Triple immunofluorescence stainings

One slide from each patient was used for triple immunofluorescence staining to be analyzed for the identification of CTCs and evaluation of KDM2B expression. A cell was characterized as CTC, when it showed positive cytokeratin (CK) expression and negative CD45 expression (expressed in PBMCs). Apart from CK-staining, cytomorphological criteria described by Meng et al. (High nuclear/cytoplasmic ratio, larger cells than white blood cells) were also applied to characterize a cell as a CTC (Meng et al., 2004). Based on the CTC morphology of every single sample, verified by the existence of CK and the absence of CD45, a second slide from the same patients was analyzed with triple immunofluorescence staining for the expression of CK, STIM1, and ORAI1. STIM1 and ORAI1 were examined simultaneously in CTCs, as these two proteins work together in the calcium signaling pathway.

The first triple immunofluorescence staining was performed using the appropriate corresponding antibodies against CK, KDM2B, and CD45. Prostate cancer patients’ cytospins were fixed with PFA 3% for 30 min, followed by permeabilization with Triton-X-100 0.5% for 10 min. Non-specific binding was avoided by blocking with 5% FBS in PBS at 4°C overnight. Cytospins were then incubated for 1 h with the anti-CD45-Alexa 647 conjugated antibody (Santa Cruz Biotechnology, Santa Cruz, CA, United States). Later, cells were further incubated for 1 h with anti-JHDM1B rabbit (KDM2B antibody, 09–864, Millipore). Alexa 488 anti-rabbit antibody was used as a secondary antibody (Life Technologies, Carlsbad, CA, United States) for 45 min. For the detection of the cytokeratins (CKs) 8/18/19, A45-B/B3 mouse antibody (Amgen, Southern Oaks, CA, United States) was used for incubation of 1 h. Alexa 555 anti-mouse antibody was used as a secondary antibody (Life Technologies, Carlsbad, CA, United States) for 45 min. Finally, samples were mounted on a Prolong antifade medium containing 4’,6-diamidino-2-phenylindole (DAPI) to observe the cell nucleus.

The second triple immunofluorescence staining was performed using the appropriate corresponding antibodies against CK, STIM1, and ORAI1. Prostate cancer patients’ cytospins were fixed and permeabilized with ice-cold acetone/methanol 9:1 (v/v) for 15 min. Non-specific binding was avoided by blocking with 5% FBS in PBS at 4°C overnight. Consequently, cells were incubated for 1 h with the anti-STIM1-Alexa 546 conjugated antibody (Santa Cruz Biotechnology, Santa Cruz, CA, United States). Cytospins were further incubated for 1 h with A45-B/B3 mouse antibody for the detection of the cytokeratins (CKs) 8/18/19 (Amgen, Southern Oaks, CA, United States). Alexa 488 anti-mouse antibody (FITC) was used as a secondary antibody (Life Technologies, Carlsbad, CA, United States) for 45 min. In the next step, cells were incubated for 1 h with the anti-ORAI1-Alexa 647 conjugated antibody (Santa Cruz Biotechnology, Santa Cruz, CA, United States). Finally, samples were mounted on a Prolong antifade medium containing 4’,6-diamidino-2-phenylindole (DAPI) to observe the cell nucleus.

PC-3 and DU-145 cells spiked in healthy volunteers’ PBMCs were used as positive and negative controls. Specifically, negative controls were prepared by omitting one of the primary antibodies and incubating the cells with the respective secondary antibodies. Each experiment included three different negative controls (for each one of the primary antibodies) and one positive for all the antibodies.

Slides from both triple immunofluorescence stainings were analyzed with the VyCAP system (VyCAP B.V., Enschede, Netherlands). The VyCAP system is an imaging system, where cytospins slides were scanned automatically using four different channels (for the first immunofluorescent staining; DAPI, CK, KDM2B, CD45, and for the second immunofluorescent staining; DAPI, CK, STIM1, ORAI1), and the corresponding frames were analyzed to identify and characterize patients’ CTCs. The VyCAP system surpasses the limitations of EpCAM-dependent methods such as losing CTCs under EMT (Adams et al., 2015; Hyun et al., 2016; Messaritakis et al., 2017; Satelli et al., 2017; Zavridou et al., 2020; Ntzifa et al., 2021). Furthermore, the corresponding frames of patients’ slides were also double-checked using the ACCEPT software (automatic software for CTCs detection, University of Twente, Enschede, Netherlands). Images were captured based on the exposure time of the negative and positive controls for each antigen. The same exposure times were applied for control samples and CTCs’ images. Finally, the identification of CTCs was performed blindly to clinical data.

### 2.5 Statistical analysis of the clinical data

Statistical tests were performed with the statistical software SPSS version 27 (IBM, Armonk, NY, United States) at the <0.05 level of significance. Progression-free survival (PFS) was defined as the time between enrolment to the study, and disease relapse or death, whatever occurred first. Overall survival (OS) was defined as the period from enrollment to the study, until death from any cause or the last time follow-up that the patient was reported alive. Statistical tests between the mean percentages of phenotypes were carried out with Wilcoxon signed-rank nonparametric tests. Correlation tests among phenotypes and clinical characteristics was performed with Spearman’s rho test. Analysis of the rest of the clinical data did not provide any further statistically significant results due to the small number of patients and the pilot nature of this study.

## 3 Results

### 3.1 KDM2B in PC-3 and DU-145 prostate cancer cell lines

**FIGURE 1:**
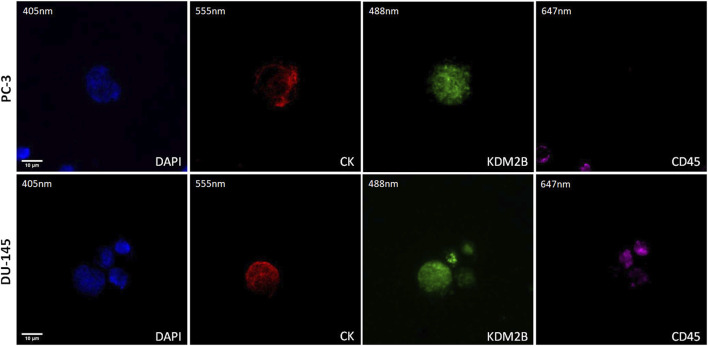
Expression of KDM2B in PC-3 and DU-145 cell lines spiked in normal donors’ PBMCs. Cells were stained for Cytokeratin (CK, red), KDM2B (green), and CD45 (purple). Nuclei (blue) were stained with DAPI. Magnification ×40. Scale bars 10 μm.

The expression of KDM2B was first visualized in cytospins of spiked PC-3 and DU-145 cancer cells with PBMCs by immunofluorescence using antibodies for CK, KDM2B, and CD45 (Figure 1). KDM2B had low cytoplasmic and mainly nuclear expression in both cell lines, in line with previous publication regarding KDM2B subcellular locations in glioma cells (Wang et al., 2018). In next step we investigated whether this expression pattern was visualized in prostate cancer patients’ CTCs.

### 3.2 KDM2B in CTCs from metastatic prostate cancer patients

**FIGURE 2:**
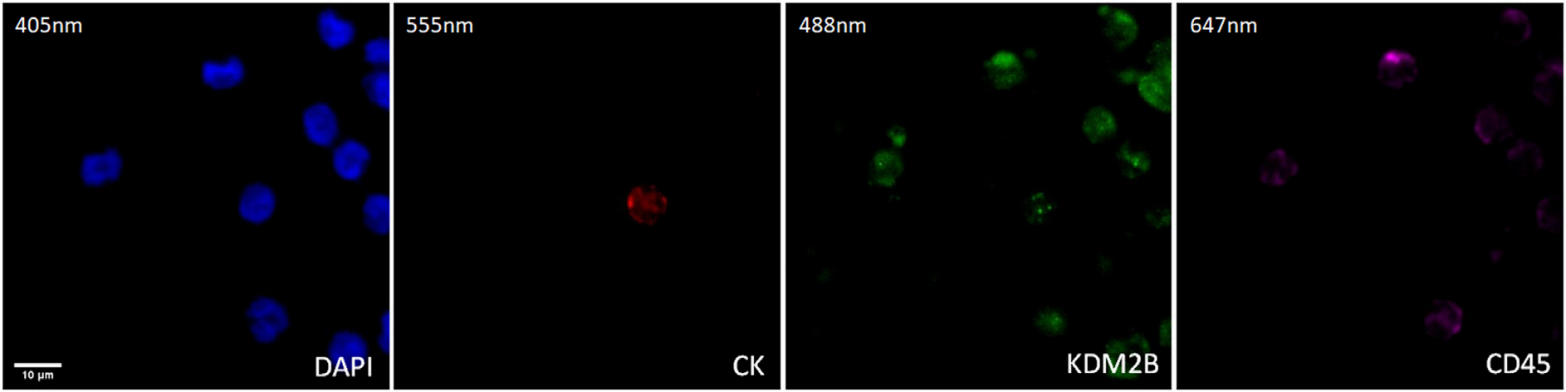
Expression of KDM2B in CTC from prostate cancer patients. Cytokeratin (CK, red), KDM2B (green), and CD45 (purple) expression. Nuclei (blue) were stained with DAPI. Magnification ×40. Scale bar 10 μm.

The expression of KDM2B in prostate cancer CTCs was examined by immunofluorescence using antibodies for CK, KDM2B, and CD45. The intracellular distribution of KDM2B was both cytoplasmic and nuclear in patients’ CTCs (Figure 2).

Thirty-two prostate cancer patients were analyzed. CTCs (CK-positive) were detected in 41% of patients (13 out of 32).

**FIGURE 3:**
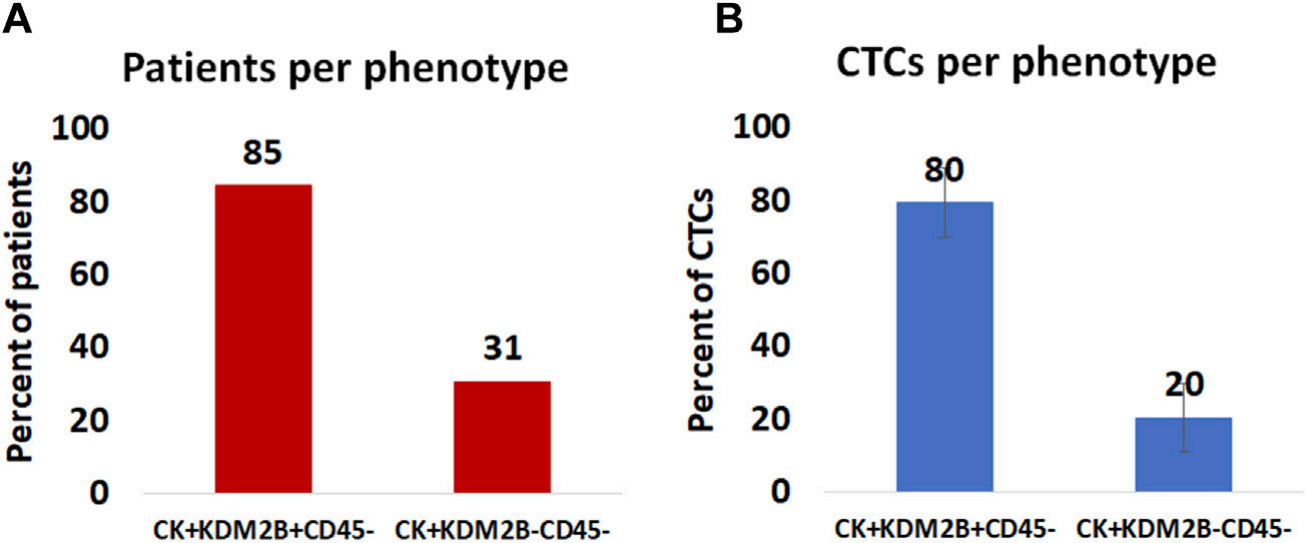
**(A)**: Percentage of patients harboring KDM2B expression in their CTCs. **(B)**: Average percentage of CTCs with the (CK+/KDM2B+/CD45–) and (CK+/KDM2B–/CD45–) phenotypes.

Among the CK-positive patients; 85% (11 out of 13) had the (CK+/KDM2B+/CD45–) phenotype, while 31% (4 out of 13) had the (CK+/KDM2B–/CD45–) phenotype (Figure 3A).

The 23% (3 out of 13) of CK-positive patients had simultaneously two phenotypes: (CK+/KDM2B+/CD45–) and (CK+/KDM2B–/CD45–). The 77% (9 out of 13) had only the (CK+/KDM2B+/CD45–) phenotype, while the 8% (1 out of 13) had only the (CK+/KDM2B–/CD45–) phenotype (Supplementary Table S1).

Regarding average percentages of total examined CTCs, 80% were classified as (CK+/KDM2B+/CD45–) and 20% as (CK+/KDM2B–/CD45–) (Figure 3B).

### 3.3 ORAI1 and STIM1 in PC-3 and DU-145 prostate cancer cell lines

**FIGURE 4:**
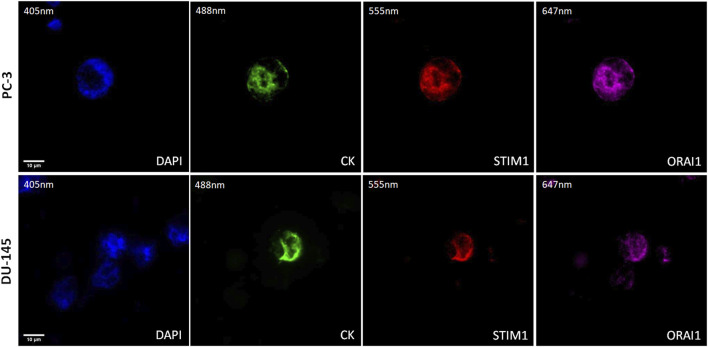
Expression of ORAI1 and STIM1 in PC-3 and DU-145 cell lines spiked in normal donors’ PBMCs. Cells were stained for Cytokeratin (CK, green), STIM1 (red), and ORAI1 (purple). Nuclei (blue) were stained with DAPI. Magnification ×40. Scale bars 10 μm.

The expression of STIM1 and ORAI1 was first evaluated in cytospins of spiked PC-3 and DU-145 cancer cells with PBMCs by immunofluorescence using antibodies against CK, STIM1, and ORAI1 (Figure 4). Both STIM1 and ORAI1 were found to be overexpressed in both prostate cancer cell lines, while in PBMCs the expression of STIM1 and ORAI1 was very low or absent, implying that both biomarkers are specific for tumor cells in the bloodstream. The next step was to investigate whether this expression pattern could be detected in prostate cancer patients.

### 3.4 ORAI1 and STIM1 in CTCs from metastatic prostate cancer patients

**FIGURE 5:**
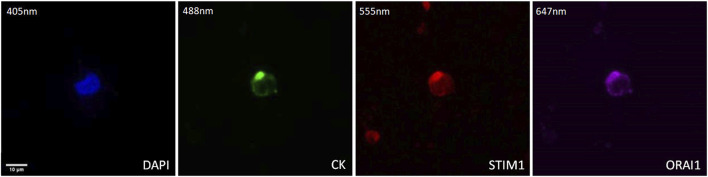
Expression of ORAI1 and STIM1 in CTCs from prostate cancer patients. Cells were stained for Cytokeratin (CK, green), STIM1 (red), and ORAI1 (purple). Nuclei (blue) were stained with DAPI. Magnification ×40. Scale bar 10 μm.

The expression of STIM1 and ORAI1 in prostate cancer CTCs was examined by immunofluorescence using antibodies for CK, STIM1, and ORAI1. Their expression was mainly cytoplasmic adjust to the plasma membrane (Figure 5).

Thirty-two prostate cancer patients were analyzed. CTCs (CK-positive) were detected in 56% of patients (18 out of 32).

**FIGURE 6:**
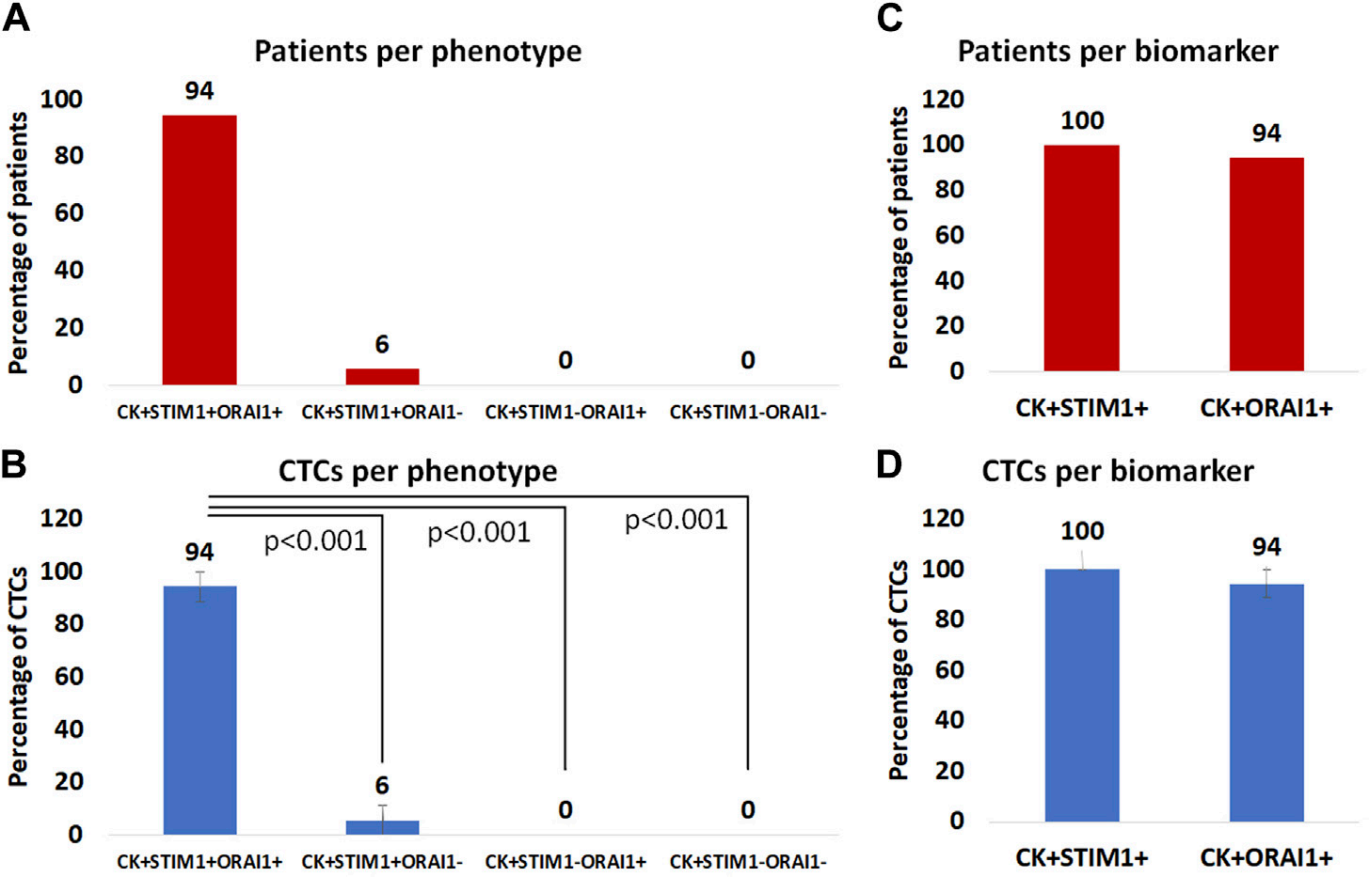
**(A)**: Percentage of patients with (CK+/STIM1+/ORAI1+), (CK+/STIM1+/ORAI1–), (CK+/STIM1–/ORAI1+) and (CK+/STIM1–/ORAI1–) phenotypes in their CTCs. **(B)**: Average percentage of CTCs with the (CK+/STIM1+/ORAI1+), (CK+/STIM1+/ORAI1–), (CK+/STIM1–/ORAI1+) and (CK+/STIM1–/ORAI1–) phenotypes. **(C)** Percentage of patients with only STIM1 or ORAI1 expression. **(D)** Average percentage of CTCs with only STIM1 or ORAI1 expression.

Among the CK-positive patients the vast majority; 94% (17 out of 18) had the (CK+/STIM1+/ORAI1+) phenotype, while only 6% (1 out of 18) had the (CK+/STIM1+/ORAI1–) phenotype. None of the patients had the (CK+/STIM1–/ORAI1+) or the (CK+/STIM1–/ORAI1–) phenotype (Figure 6A). All patients had only one detectable phenotype (Supplementary Table S2).

Regarding average percentages of total examined CTCs, 94% were classified as (CK+/STIM1+/ORAI1+) and 6% as (CK+/STIM1+/ORAI1–). None of the CTCs belonged to the following phenotypes: (CK+/STIM1–/ORAI1+) or (CK+/STIM1–/ORAI1–) (Figure 6B). As it is shown in Figure 6B there was a statistically significant (*p < 0.0001*) difference between the (CK+/STIM1+/ORAI1+) and all the other phenotypes.

Separate analysis of each biomarker revealed that all patients with identified CTCs 100% (18 of 18) were (CK+/STIM1+) and 94% (17 out of 18) were (CK+/ORAI1+) (Figure 6C).

The same percentages were observed from the analysis of the average frequency of the total observed CTCs; all CTCs were STIM1-positive, whereas 94% were ORAI1-positive (Figure 6D). None of the identified CTCs were only positive for ORAI1 and not for STIM1.

Statistical analysis revealed that the presence of (CK+/STIM1+/ORAI1–) phenotype significantly related (Spearman’s rho analysis, *p = 0.049*) with relapse in metastatic prostate cancer patients. Furthermore, the (CK+/STIM1+/ORAI1+) phenotype was related to bone metastasis (Spearman’s rho analysis, *p = 0.034*).

## 4 Discussion

CTCs have a fundamental role in metastatic dissemination by infiltrating the bloodstream and migrating to establish distant lesions (Chambers, Groom, and MacDonald 2002). They are considered the seeds of metastasis since it has been recently proved that they create cancer and promote metastasis in mice xenograft models (Baccelli et al., 2013; Hodgkinson et al., 2014). It is also widely accepted that most of the CTCs harbor both epithelial and mesenchymal properties (Kallergi et al., 2011; Nieto et al., 2016; Satelli et al., 2017; Kallergi et al., 2018; Ntzifa et al., 2021). However, the mesenchymal nature of CTCs poses a challenge to their isolation (Kallergi et al., 2011; Satelli et al., 2017; Zavridou et al., 2020). Many current isolation methods rely on epithelial markers, such as CellSearch, which is the golden standard for CTC detection particularly in metastatic breast (Cristofanilli et al., 2004), colorectal (Gazzaniga et al., 2013), and prostate cancer (Olmos et al., 2009). However, these methods fail to detect CTCs under EMT process, due to the absence of EpCAM and other epithelial antigens (Adams et al., 2015; Hyun et al., 2016; Messaritakis et al., 2017; Satelli et al., 2017; Zavridou et al., 2020; Ntzifa et al., 2021). In an older study, 34 human breast cancer cell lines were tested. Normal-like breast cancer cell lines were not identified from the CellSearch system (Sieuwerts et al., 2009). Another study in prostate cancer patients showed that identification of CTCs with an antibody against cell-surface vimentin had higher sensitivity and specificity, while in castration-resistant patients, more CTCs were identified compared to CellSearch (Satelli et al., 2017). A previous study of our group, using Ficoll density gradient and immunofluorescent staining, in patients with small cell lung cancer, showed that CK^+^/Vim^+^ CTCs (EMT CTCs), could be detected in patients whereas in the same samples, CellSearch had negative results. Furthermore, this phenotype was correlated with poor survival (Messaritakis et al., 2017). Another study examined the isolation of CTCs from 30 patients with breast and prostate cancer, through a microfiltration system (CellSieve) and the CellSearch system; showed that the CellSieve isolated more CTCs as opposed to the CellSearch, and they could be divided into five morphologically distinct subpopulations, characteristic of CTC heterogeneity (Adams et al., 2015). Overall the heterogeneous nature of CTCs is a well-described observation in terms of their morphological characteristics, protein expression patterns, and molecular profile (Polzer et al., 2014; Adams et al., 2015; Hyun et al., 2016). Therefore, new biomarkers are necessary to improve the identification rate of CTCs and to provide potential new therapeutic targets.

CTCs can persist either individually or as clusters, remaining dormant for years until they give rise to new lesions, underscoring their resistance to conventional therapies (Vishnoi et al., 2015; Matikas et al., 2022). The reason for this resistance is mainly their non-proliferating physiology making them unable to be destroyed by drugs targeting mitosis or cell proliferation. However, in castration, resistant prostate cancer patients’ chemotherapy is the main therapeutic strategy. Thus, it is extremely important the identification of new therapeutic targets in CTCs for improving survival of prostate cancer patients. It has also been proved that the enumeration of CTCs is not as important as finding the specific subpopulations which are the most aggressive subclones, related to patients’ metastasis (Kallergi et al., 2013; Satelli et al., 2016). Interestingly, recent studies showed that CTCs can be destroyed by targeted therapy, and this approach is related to patients’ survival (Georgoulias et al., 2012; Fehm et al., 2021).

On the other hand, store-operated calcium entry (SOCE), a primary mechanism for cellular calcium influx, is controlled by a store-operated calcium channel composed of ORAI1 and STIM1 proteins. The oncogenic potential of ORAI1/STIM1 has been proved, as their inhibition has been shown to suppress proliferation, migration, and invasion. Previous research from our group has demonstrated that lithium significantly enhances ORAI1 and STIM1 transcript and protein levels, along with increasing SOCE in the neurodegenerative disease Chorea-Acanthocytosis. These effects were reversed upon treatment with wogonin, an NFκB inhibitor (Sukkar et al., 2018), suggesting that this pathway holds promise for future exploration. We have also recently shown that overexpression of KDM2B in prostate cancer cells resulted in a significant upregulation of ORAI1 and Stim1 transcription. Additionally, KDM2B upregulation increased the mRNA expression of Nhe1, a Na+/H+ exchanger. Notably, ORAI1, STIM1, and Nhe1 have all been reported to be upregulated by SGK1. Previous studies have underscored the significance of SGK1 in upregulating SOCE and influencing migration and cell survival (Lang et al., 2010; Lang and Stournaras, 2013; Lang et al., 2019). In the same study, we explored the expression of KDM2B in CTCs of a small cohort of prostate cancer patients. It was interesting that 80% of the CTC-positive patients harbored KDM2B-positive CTCs. Based on the previous data the current study investigated at protein level the expression of ORAI1, STIM1, and KDM2B in CTCs from prostate cancer patients. This study confirmed the increased expression of KDM2B in CTCs from prostate cancer patients revealing that 85% of the patients harbored KDM2B-positive CTCs, which is line with the results from the smaller group of patients, implying that this expression is a common phenomenon for prostate cancer.

The method of ORAI1 and STIM1 staining was developed using PC-3 and DU-145 spiked in PBMCs cells. Interestingly as it is shown in Figure 1 the ORAI1 and STIM1 were exclusively expressed in cancer cells and not in the nearby PBMCs making them interesting biomarkers for identifying CTCs in the bloodstream. We also investigated this expression in prostate cancer patients’ CTCs, isolated with Ficoll density gradient. We found that the vast majority of prostate cancer CTCs had simultaneously both STIM1 and ORAI1 overexpression (94%). Only one patient had one CTC with STIM1 and not ORAI1 (6%) expression. None of the patients had CTCs with ORAI1 overexpression alone and none of the patients had CTCs in with absence of both STIM1 and ORAI1. In addition, all 100% of the CK-positive patients harbored CTCs with STIM1 and 94% with ORAI1 expression. The above indications are very interesting for two reasons: a) These biomarkers have extremely high expression in CTCs, higher than most of the other identified biomarkers so far, making them important potential targets for the corresponding therapeutic approaches, b) The expression of ORAI1 and STIM1 in PBMCs is very low implying that they can be interesting biomarkers for the identification of these cells in the bloodstream. These results are in line with previous publications, referring that STIM1 and ORAI1 are implicated in metastasis in prostate cancer (Xu et al., 2015; Stagno et al., 2017; Zhou et al., 2017). STIM1 overexpression has been shown to promote cell migration and EMT through TGF-β, Snail and Wnt/β-Catenin signaling in prostate cancer cell lines of PC-3 and DU-145 (Xu et al., 2015). STIM1 knockdown inhibited migration and invasion of PC-3 and DU-145 cells through the inactivation of PI3K/Akt signaling pathway while it was also proposed as a potential target against prostate cancer metastasis (Zhou et al., 2017). In a previous work of our group, a tested drug called istaroxime caused a strong inhibition of cell migration and motility in treated DU-145 cells, through the reduction of Stim1, ORAI1 transcripts, ORAI1 protein, and activated SOCE and FAK. The inhibition of cancer cell migration from istaroxime was further enhanced by blocking ORAI1 and FAK with the corresponding inhibitors (Stagno et al., 2017).

This study also revealed that the expression of the double positive phenotype (CK+STIM1+/ORAI1+) is related to bone metastasis and the (CK+STIM1+/ORAI1–) phenotype to disease recurrence. This is also in line with a previous study showing that STIM1 mediates cell invasion and bone metastasis in prostate cancer patients (Zhou et al., 2023). In the current study, the correlation of this phenotype with bone metastasis underlies the important role of these cells and the necessity of their enumeration and characterization in every patient in the precision medicine approach.

## Data Availability

The original contributions presented in the study are included in the article/Supplementary Material, further inquiries can be directed to the corresponding author.

## Abbreviations

SOCE, store operated Ca^2+^ entry; CTCs, circulating tumor cells; SGK1, glucocorticoid-regulated kinase 1.
