# Supplementary material for: STIM1, ORAI1, and KDM2B in circulating tumor cells (CTCs) isolated from prostate cancer patients
Source: Front Cell Dev Biol. 2024 Jun 5;12:1399092. doi: 10.3389/fcell.2024.1399092 (PMC11188415; doi:10.3389/fcell.2024.1399092)
Supplement: Supplementary file 1 [file Table1.DOCX]

Supplementary Material

Table S1. Results for CTCs in patients with metastatic prostate cancer from triple immunofluorescent staining CK/KDM2B/CD45.

| Patient number | Total CK+ cells (CTCs) | CK+/KDM2B+/CD45- | CK+/KDM2B-/CD45- |
| --- | --- | --- | --- |
| 1 | 0 | 0 | 0 |
| 2 | 2 | 1 | 1 |
| 3 | 1 | 1 | 0 |
| 4 | 1 | 1 | 0 |
| 5 | 2 | 0 | 2 |
| 6 | 10 | 4 | 6 |
| 7 | 0 | 0 | 0 |
| 8 | 0 | 0 | 0 |
| 9 | 9 | 4 | 5 |
| 10 | 1 | 1 | 0 |
| 11 | 0 | 0 | 0 |
| 12 | 0 | 0 | 0 |
| 13 | 0 | 0 | 0 |
| 14 | 0 | 0 | 0 |
| 15 | 1 | 1 | 0 |
| 16 | 0 | 0 | 0 |
| 17 | 0 | 0 | 0 |
| 18 | 0 | 0 | 0 |
| 19 | 0 | 0 | 0 |
| 20 | 1 | 1 | 0 |
| 21 | 3 | 3 | 0 |
| 22 | 2 | 2 | 0 |
| 23 | 1 | 1 | 0 |
| 24 | 0 | 0 | 0 |
| 25 | 0 | 0 | 0 |
| 26 | 0 | 0 | 0 |
| 27 | 0 | 0 | 0 |
| 28 | 0 | 0 | 0 |
| 29 | 0 | 0 | 0 |
| 30 | 0 | 0 | 0 |
| 31 | 0 | 0 | 0 |
| 32 | 1 | 1 | 0 |

Table S2. Results for CTCs in patients with metastatic prostate cancer from triple immunofluorescent staining CK/STIM1/ORAI1.

| Patient number | Total CK+ cells (CTCs) | CK+/STIM1+/ORAI1+ | CK+/STIM1-/ORAI1+ | CK+/STIM1+/ORAI1- | CK+/STIM1-/ORAI1- |
| --- | --- | --- | --- | --- | --- |
| 1 | 0 | 0 | 0 | 0 | 0 |
| 2 | 0 | 0 | 0 | 0 | 0 |
| 3 | 0 | 0 | 0 | 0 | 0 |
| 4 | 0 | 0 | 0 | 0 | 0 |
| 5 | 0 | 0 | 0 | 0 | 0 |
| 6 | 1 | 1 | 0 | 0 | 0 |
| 7 | 1 | 1 | 0 | 0 | 0 |
| 8 | 1 | 0 | 1 | 0 | 0 |
| 9 | 0 | 0 | 0 | 0 | 0 |
| 10 | 1 | 1 | 0 | 0 | 0 |
| 11 | 1 | 1 | 0 | 0 | 0 |
| 12 | 0 | 0 | 0 | 0 | 0 |
| 13 | 0 | 0 | 0 | 0 | 0 |
| 14 | 1 | 1 | 0 | 0 | 0 |
| 15 | 1 | 1 | 0 | 0 | 0 |
| 16 | 0 | 0 | 0 | 0 | 0 |
| 17 | 0 | 0 | 0 | 0 | 0 |
| 18 | 1 | 1 | 0 | 0 | 0 |
| 19 | 0 | 0 | 0 | 0 | 0 |
| 20 | 1 | 1 | 0 | 0 | 0 |
| 21 | 1 | 1 | 0 | 0 | 0 |
| 22 | 1 | 1 | 0 | 0 | 0 |
| 23 | 2 | 2 | 0 | 0 | 0 |
| 24 | 1 | 1 | 0 | 0 | 0 |
| 25 | 0 | 0 | 0 | 0 | 0 |
| 26 | 0 | 0 | 0 | 0 | 0 |
| 27 | 2 | 2 | 0 | 0 | 0 |
| 28 | 3 | 3 | 0 | 0 | 0 |
| 29 | 0 | 0 | 0 | 0 | 0 |
| 30 | 1 | 1 | 0 | 0 | 0 |
| 31 | 1 | 1 | 0 | 0 | 0 |
| 32 | 2 | 2 | 0 | 0 | 0 |
